# Universal Image Restoration with Text Prompt Diffusion

**DOI:** 10.3390/s24123917

**Published:** 2024-06-17

**Authors:** Bing Yu, Zhenghui Fan, Xue Xiang, Jiahui Chen, Dongjin Huang

**Affiliations:** Shanghai Film Academy, Shanghai University, Shanghai 200072, China; zhenghui_fan@shu.edu.cn (Z.F.); 22723302@shu.edu.cn (X.X.); jiahui_chen@shu.edu.cn (J.C.); djhuang@shu.edu.cn (D.H.)

**Keywords:** image restoration, diffusion model, text prompt

## Abstract

Universal image restoration (UIR) aims to accurately restore images with a variety of unknown degradation types and levels. Existing methods, including both learning-based and prior-based approaches, heavily rely on low-quality image features. However, it is challenging to extract degradation information from diverse low-quality images, which limits model performance. Furthermore, UIR necessitates the recovery of images with diverse and complex types of degradation. Inaccurate estimations further decrease restoration performance, resulting in suboptimal recovery outcomes. To enhance UIR performance, a viable approach is to introduce additional priors. The current UIR methods have problems such as poor enhancement effect and low universality. To address this issue, we propose an effective framework based on a diffusion model (DM) for universal image restoration, dubbed ETDiffIR. Inspired by the remarkable performance of text prompts in the field of image generation, we employ text prompts to improve the restoration of degraded images. This framework utilizes a text prompt corresponding to the low-quality image to assist the diffusion model in restoring the image. Specifically, a novel text–image fusion block is proposed by combining the CLIP text encoder and the DA-CLIP image controller, which integrates text prompt encoding and degradation type encoding into time step encoding. Moreover, to reduce the computational cost of the denoising UNet in the diffusion model, we develop an efficient restoration U-shaped network (ERUNet) to achieve favorable noise prediction performance via depthwise convolution and pointwise convolution. We evaluate the proposed method on image dehazing, deraining, and denoising tasks. The experimental results indicate the superiority of our proposed algorithm.

## 1. Introduction

High-quality images exhibit clear texture details and more realistic colors, not only enhancing visual experiences but also facilitating subsequent image processing and analysis. However, image quality is affected during processes such as acquisition, transmission, and storage, making it challenging to obtain clear, high-quality images. The various types of degradation of images (such as blur, noise, raindrops, and haze) not only significantly impact visual perception but also pose difficulties and challenges for subsequent applications of the images. Therefore, restoring degraded images to high-resolution images while preserving as much information as possible, and recovering their color and texture, holds significant research significance.

Given that there are multiple degradation types, single-task image restoration methods [1,2,3,4,5,6,7,8,9,10] involve training a single-task model for each type of degradation. While such an approach may yield favorable metrics for individual tasks, its applicability to complex real-world scenarios is challenging. Additionally, if there is a shift in the degradation type or corruption ratio, the model’s performance could become unsatisfactory. This dissatisfaction arises due to the misalignment between the actual scenario encountered and the previously chosen parameters for either model construction or training. More recently, many works have achieved universal image restoration by training a learning-based model to be effectively capable of recovering images from various types of degradation. Methods can be roughly divided into two categories [11]: The first category comprises methods [12,13,14,15,16] that model image restoration tasks as linear inverse problems, using pretrained diffusion models as generative priors to solve any linear inverse problem. However, these methods require a precise definition of a function for each specific type of degradation. The second category comprises methods [11,17,18,19,20,21] that explicitly or implicitly train a degradation-type classifier in an end-to-end manner, using it as the foundation for image restoration. However, they lack the ability to generate missing or deteriorated details in images [11].

In summary, an essential aspect of enhancing image restoration lies in effectively modeling degradation, especially in intricate application contexts. However, most methods heavily rely on low-quality (LQ) image features for restoration guidance, which is challenging and limits restoration performance. In cases of severe image degradation, the degradation process may lead to the loss of essential feature information from the original image, making it challenging for the model to accurately reconstruct the original image. The introduction of additional prior information can effectively enhance the performance of image restoration. Recently, text prompts have attracted considerable attention in various fields such as image segmentation [22], image generation [23,24], and image editing [25]. Inspired by this, we incorporate text prompts as prior information for image restoration tasks.

In this paper, we propose a framework that utilizes a text prompt corresponding to a low-quality image to assist the diffusion model in restoring the image. Firstly, we use the visual language model Minigpt-4 [26] to generate corresponding textual descriptions for high-resolution images in the dataset. We utilize text prompts to represent the image’s semantic content to provide additional prior information. Considering that the LR image can provide the majority of low-frequency [27] and semantic information related to the content [23], we utilize DA-CLIP [18] to extract features related to the degradation type from the image and perform classification, facilitating the universal restoration of different degradation types. Firstly, we input the textual caption into the pretrained CLIP text encoder [28] to obtain the text encoding. Subsequently, the LQ image is fed into the pretrained DA-CLIP image controller to obtain the image degradation embedding. We then combine the text encoding and degradation embedding, followed by prompt encoding, to obtain the complete text prompt. In addition, to enhance the performance of the diffusion model in image restoration tasks, we made improvements to its denoising network. Inspired by ConvMixer [29] and ConvNeXtV2 [30], we designed a novel module for the denoising network that functions as both an encoder and a decoder. We further propose a network, efficient text prompt diffusion image restoration (ETDiffIR), to realize the text prompt universal image restoration (UIR). ETDiffIR utilizes an advanced score-based diffusion model [31]. Overall, our main contributions can be summarized as follows:

- We proposed a text prompt diffusion model to solve the universal image restoration problem. To the best of our knowledge, this is the first attempt to incorporate text prompts into universal image restoration.
- We pioneered an effective denoising network for diffusion-based image restoration. By introducing text prior information into the diffusion model using an efficient restoration block (ERB), ETDiffIR can achieve excellent image restoration results.
- We constructed a combined dataset containing three different degradation types and generated synthetic captions using the visual-language model Minigpt-4, resulting in a high-quality dataset comprising paired text and images.

## 2. Related Work

### 2.1. Universal Image Restoration

While single-task image restoration methods [1,2,3,4,5,32,33,34,35] have matured over time, universal image restoration methods are currently still in the early stages of development. Universal image restoration refers to the use of a single model to handle various types of degradation, also known as “all-in-one” image restoration. Universal image restoration methods can be broadly categorized into two categories: methods based on unsupervised generation priors and methods based on end-to-end training.

Using pretrained diffusion models as generative priors [12,13,14,15,16] for image restoration has become a popular approach in recent times. These types of methods model image restoration as a linear inverse problem. Kawar et al. [12], building upon the use of a diffusion model prior, introduced the singular value decomposition of the degradation operator during the inverse diffusion process to obtain restoration results. Similarly, Wang et al. [13] refined only the null space content during the inverse diffusion process, obtaining diverse results that achieved both data consistency and realism. Garber et al. [15] proposed a guided technique based on preprocessing, which reduces the number of iterations in the inverse diffusion process and enhances robustness. The mentioned methods require a manually defined precise degradation function for each degradation type and are limited to linear degradation.

The second category of methods is based on end-to-end learning, typically utilizing an explicitly or implicitly embedded degradation classifier within the network to determine the degradation type of the image, guiding the image restoration process [11,17,18,19,20,21]. For example, Li et al. [17] designed a contrastive learning-based encoder that leverages the consistency among images with the same type of degradation and the inconsistency present among images with different types of degradation to learn degradation representations. Chen et al. [21] employed knowledge distillation to obtain a universal image restoration model from multiple image restoration networks specializing in different degradation types. Jiang et al. [11] designed a blind image quality assessment module that automatically detects and identifies the degradation type of an image, guiding the diffusion model in image restoration. Zhang et al. [20] proposed a general image restoration method based on principal component analysis. This method established a corresponding prior center for each type of degradation and constructed task-oriented centers as single-component centers through learnable principal component analysis. Luo et al. [18] designed an image controller based on CLIP [28]. Through contrastive learning, the controller outputs degradation features that match the input image’s degradation characteristics, resulting in a natural classifier for different degradation types. More recently, Yan et al. [36] fine-tuned language models to identify and restore different types of degradation through user interaction. However, many methods still face challenges in terms of reconstruction quality. Our approach utilizes scene descriptions as additional priors to enhance image reconstruction under severe degradation.

### 2.2. Diffusion-Based Restoration

Diffusion models employ a fixed Markov chain to optimize the change boundary of the likelihood function, and they have recently gained increasing attention because of their outstanding performance in generative tasks [23]. In IR tasks, the application of diffusion models is still in its early stages. Xia et al. [37] utilized Transformer blocks to simulate long-range dependencies for noise prediction, achieving effective image reconstruction. Li et al. [38] introduced residual prediction into a diffusion model for image SR. Luo et al. [31] proposed the concept of an averaging equation to simulate the image degradation process, concurrently achieving a faster diffusion process. However, image restoration based on diffusion models often relies on a complex network to predict noise, which affects the efficiency of the model in practical applications. To address this, the proposed ETDiffIR utilizes a lightweight network, ERUNet, to predict noise, achieving satisfactory results.

## 3. Method

To enhance reconstruction performance in image restoration tasks, based on stochastic differential equations (SDEs), we design a text-conditioned diffusion model suitable for universal image restoration. Given a degraded image and a textual description of the scene in that image, we use the diffusion model to generate a high-quality (HQ) image. We train the diffusion model on a synthetic dataset of image–text pairs. In the following sections, we describe our data processing procedure and the main architecture of the model.

### 3.1. Preliminary

**Figure 1:**
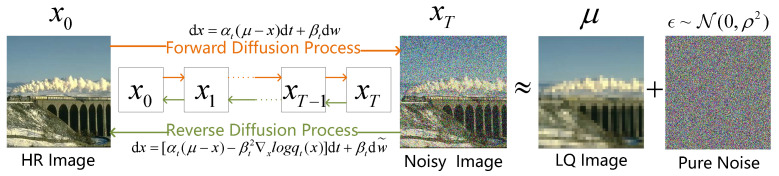
An overview of the forward diffusion process and the reverse diffusion process using mean-reverting stochastic differential equations. The forward diffusion process simulates the degradation of an HQ image $x_{0}$ into an LQ image μ via diffusion $x_{0}$ towards μ+ϵ.

Here, we describe the main components of the diffusion model relevant to our process. We adopt a mean-reverting stochastic differential equation (SDE) [31] to define the diffusion process. Given input data $x_{0}∼q$ sampled from distribution *q*, after *T* time steps of increasing noise in the forward diffusion process, $x_{0}$ is transformed into a noisy image $x_{T}$. The high-quality image $I_{HQ}$ is defined as $x_{0}$. As shown in Figure 1, SDE can simulate the degradation process from an HQ image to an LQ image by approximating $x_{T}$ as a combination of the LQ image μ and pure noise ϵ. Specifically, the forward diffusion process can be described as

(1) $$dx=\alpha_{t}\left(\mu -x\right)dt+\beta_{t}dw.$$

Here, $\alpha_{t}$ and $\beta_{t}$ are two time-dependent parameters, controlling the mean-reversion speed and the stochastic volatility, respectively. *w* represents the standard Wiener process. We set $\beta_{t}^{2}/\alpha_{t}=2\rho^{2}$ to ensure a closed-form solution for Equation (1), where $\rho^{2}$ represents the stationary variance. Given any $x_{0}$ and time step t∈[0,T], the corresponding intermediate state $x_{t}$ can be expressed by the solution of Equation (1) as follows:

(2) $$\begin{array}{cc} \; & x_{t}=\mu +\left(x_{0}-\mu \right)e^{-{\bar{\alpha }}_{t}}+\int_{0}^{t}\beta_{z}e^{-{\bar{\alpha }}_{t}}dw\left(z\right),\\ \; & x_{t}∼q_{t}\left(x\right)=N\left(x_{t}|u_{t}\left(x\right),v_{t}\right), \end{array}$$

where ${\bar{\alpha }}_{t}$ is defined to be equal to $\int_{0}^{t}\alpha_{z}dz$, and $u_{t}=\mu +\left(x_{0}-\mu \right)e^{-{\bar{\alpha }}_{t}}$ and $v_{t}=\rho^{2}\left(1-e^{-2{\bar{\alpha }}_{t}}\right)$ are the mean and variance of this Gaussian distribution, respectively. When t→∞, $u_{t}$ converges to μ and $v_{t}$ converges to $\rho^{2}$.

In the inverse diffusion process, the reversal of the process is achieved by iteratively recovering a signal from $x_{T}$. With the reversed-time SDE [39], the reverse diffusion process can be described as

(3) $$dx=\left[\alpha_{t}\left(\mu -x\right)-\beta_{t}^{2}\nabla_{x}logq_{t}\left(x\right)\right]dt+\beta_{t}d\tilde{w}.$$

The score $\nabla_{x}logq_{t}\left(x\right)$ of the marginal distribution at time step *t* is the only unknown in the inference phase. Since the HQ image $x_{0}$ is available during the training process, we can train a neural network to predict the unknown score. During the training process, the ground-truth score can be represented as

(4) $$\nabla_{x}logq_{t}\left(x|x_{0}\right)=-\frac{x_{t}-u_{t}\left(x\right)}{v(t)}.$$

Furthermore, if $x_{t}$ is reparameterized as $x_{t}=u_{t}\left(x\right)+\sqrt{v_{t}}\epsilon_{t}$, where $\epsilon_{t}$ is noise that follows a standard normal distribution N(0,I), the ground-truth score can be expressed as a noise term using

(5) $$\nabla_{x}logq_{t}\left(x|x_{0}\right)=-\frac{\epsilon_{t}}{\sqrt{v_{t}}}.$$

Since noise $\epsilon_{t}$ is the only unknown parameter, we only need to train a conditional time-dependent noise prediction network ${\hat{f}}_{\phi }$ to predict the noise. Similar to DDPM [40], the training objective for this noise prediction network can be expressed as

(6) $$L\left(\phi \right)=\Sigma_{t=0}^{T}\gamma_{t}E[∥{\hat{f}}_{\phi }\left(x_{t},\mu ,t\right)∥],$$

Here, $\gamma_{t}$ is a positive weight.

### 3.2. Overview

**Figure 2:**
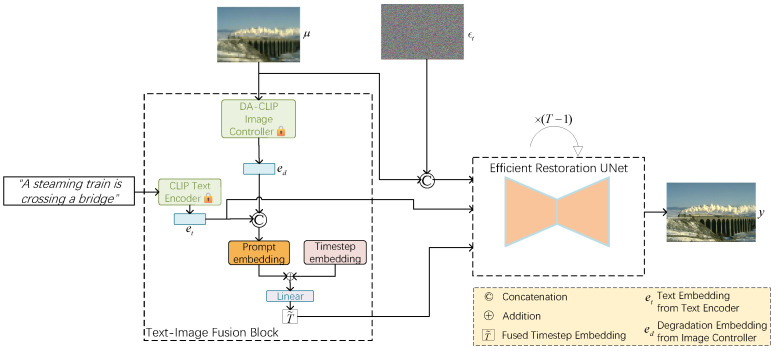
The overall architecture of our proposed ETDiffIR. It comprises a text–image fusion block (TIFB) and ERUNet for noise prediction. The TIFB incorporates a pretrained CLIP text encoder and a pretrained DA-CLIP image controller, with their weights frozen during training.

Figure 2 illustrates the architecture of our proposed ETDiffIR. To achieve a better condition for the noise prediction of the diffusion model, we introduce conditional augmentation in the input section. The LQ image μ is concatenated with noise image $\mu_{t}$ (t∼[1,T]) as the input to the ERUNet. The caption *c* is transformed into embedding by the text encoder. The image controller predicts the degradation features from the LQ image μ. ETDiffIR takes the LQ image $\mu \in R^{C_{in}\times H_{in}\times W_{in}}$ and the corresponding textual caption *c* as inputs, and it outputs the restored image $I_{r}\in R^{C_{out}\times H_{out}\times W_{out}}$. We designed a text–image fusion block (TIFB), which integrates the caption to enhance the restoration effect and extracts damage-type-related information to guide the diffusion model in image restoration. Specifically, the TIFB takes the LQ image μ, the corresponding textual caption *c*, and time step *t* as the input, and then it generates a fused time step embedding using the following formula:

(7) $$\tilde{t}=TIFB\left(\mu ,c,t\right).$$

Here, TIFB(·) represents TIFB. Subsequently, the LQ image μ, caption *c*, and fused time step embedding $\tilde{t}$ are fed into a conditional time-aware network, ${\hat{f}}_{\phi }$, aiming to obtain pure noise:

(8) $${\hat{\epsilon }}_{t}={\hat{f}}_{\phi }\left(\mu^{⌢}\epsilon_{t},c,\tilde{t}\right).$$

Here, ⌢ represents concatenation. We use an efficient restoration U-shaped network (ERUNet) to predict the noise, and, finally, we optimize ${\hat{f}}_{\phi }$ until convergence.

### 3.3. Text–Image Fusion Block

To leverage text information when recovering degraded images, we encode the caption and then integrate it into the diffusion model. Additionally, to make the model adaptable to different degradation types, a degradation-type classifier is required to encode the degradation type of the image. Our text–image fusion block (TIFB) integrates text encoding and degradation encoding into the time step encoding of the diffusion model, facilitating the restoration of images with different degradation types.

Pretrained language models have strong text comprehension capabilities. Therefore, we use a pretrained text encoder to build our network. As shown in Figure 2, our TIFB uses a pretrained CLIP text encoder to encode input caption *c* into a caption embedding, $e_{t}$. It also uses a pretrained DA-CLIP [18] image controller to discern the degradation features of the LQ image and obtain the degradation embedding $e_{d}$. The captions are processed by the text encoder of CLIP, which is a ViT-B/32 model, producing a 512-dimensional representation vector. This step encodes textual information into image-level features that align with high-definition image content, optimizing the restoration results with additional semantic signals. The LQ images are passed through the DA-CLIP image controller, also generating a 512-dimensional representation vector $e_{d}$. The DA-CLIP image controller is derived from a fine-tuned CLIP image encoder, and its output vectors include image content features and image degradation features. Then, we concatenate $e_{t}$ and $e_{d}$ and embed them as prompts. The time step *t* of the diffusion model is encoded as a time embedding $t_{emb}$. Finally, we add the prompt to the $t_{emb}$ of the diffusion model and pass them through a linear layer to obtain the fused time embedding $\tilde{t}$. The prompt, which combines caption embeddings and degradation embeddings, can facilitate degradation-type classification in universal image restoration, thereby improving the restoration results.

### 3.4. Efficient Restoration Block

**Figure 3:**
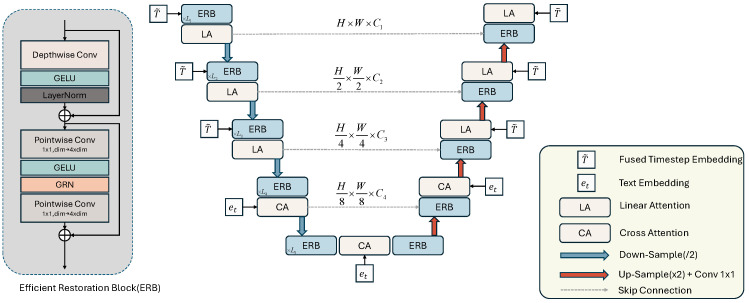
Illustration of the efficient restoration U-shaped network (ERUNet).

To efficiently extract contextual information and reduce the parameter count, inspired by ConvNeXtV2 [30] and ConvMixer [29], we designed an efficient restoration block (ERB). The core of the ERB is depthwise separable convolution (DSC), which is the combination of depthwise convolution and pointwise convolution, which are well known due to Xception [41] and MobileNet [42]. Depthwise convolution independently convolves each input channel using filters specific to each channel. Pointwise convolution combines the results of the depthwise convolution through pointwise convolution, utilizing a 1 × 1 convolution kernel. The separated depthwise convolution is used to extract the spatial dimension information, and the pointwise convolution is used to amalgamate the features learned by different channels to form the final output. DSC is commonly employed in lightweight model design to reduce the number of parameters and calculation quantity. Figure 3 details the structure of our proposed ERB: we employ depthwise convolution with a large kernel size to extract global information for each channel, followed by residual connections. After depthwise convolution, we apply two pointwise convolutions with an inverted bottleneck design to fully fuse spatial and channel information. The inverted bottleneck design has been explored using ConvNeXt [43]. The expanded hidden dimensions allow for a comprehensive fusion of the globally extracted spatial information through depthwise convolution. Additionally, we apply GELU activation and post-activation global response normalization (GRN) [30] after each convolution to enhance channel contrast and selectivity. Our ERB is defined as follows:

(9) $$f_{l}^{\prime }=LN(GELU(DW\left(f_{l-1}))\right)+f_{l-1},$$

(10) $$f_{l}^{\prime \prime }=GRN(GELU(PW\left(f_{l}^{\prime }))\right),$$

(11) $$f_{l}=PW\left(f_{l}^{\prime \prime }\right)+f_{l}^{\prime },$$

Here, $f_{l}$ represents the output feature map of layer *l* in the ERB, LN represents the layer normalization layer, GLUE represents the GLUE activate function, DW represents the depthwise convolution, and PW represents the pointwise convolution.

### 3.5. Efficient Restoration U-Shaped Network for Noise Prediction

As shown in Figure 3, we introduce the ERB into the encoder–decoder part of the efficient restoration U-shaped network (ERUNet). Compared to complex UNet architectures that include self-attention mechanisms, this lightweight design significantly reduces the computational complexity of the network. In tasks related to image restoration, the majority of the image pixels are known [44]. Therefore, large models with a high computational complexity are inefficient for IR tasks. Our ERUNet achieves optimal performance while remaining relatively lightweight.

As illustrated in Figure 3, our proposed ERUNet consists of a total of five layers from the top layer to the bottom layer, and it is divided into the encoder stage and the decoder stage. In the encoder stage, we use the ERB to extract multiscale global context information. After each ERB, a linear attention module is placed to capture long-range information, enhancing the model’s understanding of the overall structure of the feature. Then, a vanilla convolution with a stride of 2 is used to downsample the feature map. Since the bottom of the U-shaped structure retains high-level abstract features from the input, providing additional contextual information for the decoder, we utilize a cross-attention mechanism [45] to map the text embedding $e_{t}$ to the intermediate layer of UNet, enhancing the guidance of the caption in image restoration. In the decoder stage, we utilize multiple ERBs to decode features, and a nearest-neighbor interpolation is used to upsample the features, followed by a 3 × 3 convolution operation to adjust the number of channels. The numbers of ERBs in the encoder and decoder are denoted as $\left[a_{1},a_{2},a_{3},a_{4}\right]$ and $\left[b_{1},b_{2},b_{3},b_{4}\right]$, respectively. Additionally, we place *k* ERBs in the bottom layer of the ERUNet.

### 3.6. Optimization and Inference

While Equation (6) provides a straightforward optimization objective for the ERUNet, training can become unstable when the diffusion model encounters complex image degradation. This is because predicting instantaneous noise at a given moment is challenging. Following previous work [31], we employ a maximum likelihood learning strategy to alter the optimization objective. To train the ERUNet, we optimize the following function:

(12) $$L\left(\phi \right)=\Sigma_{t=0}^{T}\gamma_{t}E[∥\overset{\mathrm{reversed}\;x_{t-1}}{\overbrace{x_{t}-{\left(dx_{t}\right)}_{{\hat{f}}_{\phi }}}}-x_{t-1}^{*}∥].$$

Here, $x_{t-1}^{*}$ represents the theoretical state reversed from $x_{t}$. The closed form of $x_{t-1}^{*}$ can be represented by the following equation:

(13) $$x_{t-1}^{*}=\frac{1-e^{-2{\bar{\alpha }}_{t-1}}}{1-e^{-2{\bar{\alpha }}_{t}}}e^{-\alpha_{t}^{\prime }}\left(x_{t}-\mu \right)+\frac{1-e^{-2\alpha_{t}^{\prime }}}{1-e^{-2{\bar{\alpha }}_{t}}}e^{-\bar{\alpha_{t-1}}}\left(x_{0}-\mu \right)+\mu .$$

For the proof, please refer to [31]. Briefly, we replace the distance between the predicted state and the ideal state with the distance between the predicted noise and the true noise. Given that the majority of pixels are known to be in the reversed state, this approach helps to stabilize the optimization process. In the inference phase, the pretrained ${\hat{f}}_{\phi }$ samples the initial state $x_{t}$, and the Euler–Maruyama method [46] iteratively solves the SDE. Algorithms 1 and 2 describe the training and inference processes of our ETDiffIR, respectively.
**Algorithm 1** Training of ETDiffIRInput:LR image $\mu =I_{LR}$, HR image $x_{0}=I_{HR}$, text caption *c*, total step *T*.1: Initialization: Random sample $\epsilon_{t}∼N\left(0,\rho^{2}\right)$, t∈[0,T], T=100.2: **repeat**3:    $\tilde{t}=\mathrm{TIFB}\left(\mu ,c,t\right)$;                              ▷Enhance4:    ${\hat{\epsilon }}_{t}={\hat{f}}_{\phi }\left(I_{t},c,\tilde{t}\right)$;                           ▷Predict noise5:    $dx=\left[\alpha_{t}\left(\mu -x\right)-\beta_{t}^{2}\nabla_{x}logq_{t}\left(x\right)\right]dt+\beta_{t}d\tilde{w}$;    ▷Substitute score into Equation (6)6:    $L\left(\phi \right)=\Sigma_{t=0}^{T}\gamma_{t}E[∥\overset{\mathrm{reversed}\;x_{t-1}}{\overbrace{x_{t}-{\left(dx_{t}\right)}_{{\hat{f}}_{\phi }}}}-x_{t-1}^{*}∥]$;                    ▷Loss7:    $\nabla_{\phi }L$;                               ▷Gradient descent8:**until** converged

**Algorithm 2** Inference of ETDiffIRInput: LR image $\mu =I_{LR}$, text caption *c*, total step *T*. **Output:** The restored image $I_{HR}$.
1: Initialization: Random sample $x_{T}∼N\left(0,\rho^{2}\right)$, $f_{\phi }$ is the pretrained ERUNet, *T* = 100. EM is Euler-Maruyama method.2: **for** t=T **to** 1 **do**3:    $\tilde{t}=\mathrm{TIFB}\left(\mu ,c,t\right)$;                              ▷Enhance4:    ${\hat{\epsilon }}_{t}={\hat{f}}_{\phi }\left(x_{t},\mu ,c,\tilde{t}\right)$                             ▷Predict noise5:    $dx=\left[\alpha_{t}\left(\mu -x\right)-\beta_{t}^{2}\nabla_{x}logq_{t}\left(x\right)\right]dt+\beta_{t}d\tilde{w}$;    ▷Substitute score into Equation (6)6:    $x_{t-1}=x_{t}-\mathrm{EM}\left(dx_{t}\right)$;                            ▷Reverse SDE7: 
**end for**
8: $I_{HR}=x_{0}$;


## 4. Experiments

In Section 4.1, Section 4.2 and Section 4.3, we introduce the experimental settings, including the experimental details, hardware specifications, datasets, and evaluation metrics. The performance comparisons and ablation experiments are specifically described in Section 4.4, Section 4.5 and Section 4.6, respectively.

### 4.1. Datasets

To validate the effectiveness of the ETDiffIR, we evaluated our method on three popular image restoration tasks: image denoising, image deraining, and image dehazing. We trained and evaluated the model separately in a universal setting and a single-task setting.

For the universal setting, following Airnet, we used WED [47], BSD400 [48], CBSD68 [49], Rain100L [50], and SOTS [51] for training and testing. For the image denoising task, we used the mixed datasets of WED and BSD400. The WED dataset contains 4744 high-quality training images, while the BSD400 dataset contains 400 training images. We added Gaussian noise with a variance of 50 to clean images from these datasets to obtain noisy images. The CBSD68 dataset was used for testing. For the image deraining task, the Rain100L dataset was employed, which comprises 200 clean–rainy image pairs for training and 100 image pairs for testing. For the image dehazing task, the SOTS dataset was used, consisting of 72,135 training images and 500 testing images. Finally, to train a unified model in the general setting, we combined the above datasets and trained a single model, which was then evaluated on multiple tasks.

For the single-task setting, we trained and evaluated the model using more challenging datasets and compared it against advanced methods for each task. The Rain100H [50] dataset was used for image deraining in the single-task setting. This dataset comprises 1000 clean–rainy image pairs for training and 100 image pairs for testing. For the image dehazing task, the RESIDE-6k [51] dataset was utilized, consisting of 6000 training images and 1000 testing images. For the image denoising task, we continued to train on the WED and BSD400 datasets, with CBSD68 for testing.

**Figure 4:**
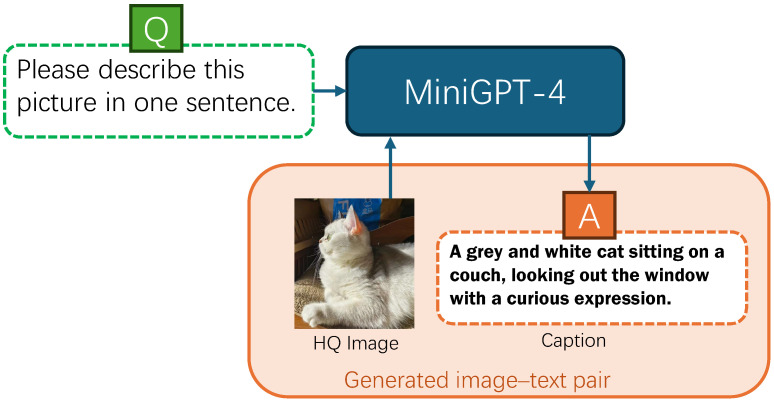
An example of image–text pair generation using Minigpt-4.

To train the ETDiffIR, we used the advanced visual language model MiniGPT-4 to generate synthetic captions for the HQ images in the dataset. Following TFRGAN [52], we also used the captions corresponding to the HQ images as prompts during testing. Since the inputs were high-resolution images, the generated captions were accurate. As shown in Figure 4, we directly used these captions to generate the text–image pairs.

### 4.2. Implementation Details

In this research, we designed an efficient diffusion probabilistic model guided by text to recover visually pleasing HQ images from LQ images. The network is designed to receive 3-channel image inputs and tokenized text inputs. During training, we performed data preprocessing by reading text–image pairs from the dataset. The images were cropped to 256 × 256 pixels patches and the corresponding captions are encoded into 512-dimensional tokens using CLIP ViT-B/32 text encoder. To enhance the model’s robustness, we performed random horizontal and vertical flips on the images for data augmentation. To ensure that our model has an appropriate size, the depth of our ERUNet is set to 4 layers. The inner-channel number in the ERUNet is set to 64. The number of ERBs in each depth of $\left[a_{1},a_{2},a_{3},a_{4}\right]$ and $\left[b_{1},b_{2},b_{3},b_{4}\right]$ is set to [2, 2, 2, 2] and [1, 1, 1, 1], respectively. There are two ERBs in the bottom layer of the ERUNet. Our experiments were carried out on a Linux server running Ubuntu 22.04. The CPU version was Intel Xeon w7-3465X, and two NVIDIA RTX A5000 graphics cards were used. The PyTorch version was 2.1.1, and the Python version was 3.8.18. We performed 600,000 iterations with a batch size of 12. The initial learning rate was set to 2 × $10^{-4}$. The cosine annealing learning rate adjustment strategy was employed. We utilized the AdamW optimizer, with $\beta_{1}$ set to 0.9 and $\beta_{2}$ set to 0.99. The total time steps for the diffusion process were set to T = 100.

### 4.3. Metrics

In this paper, we use five metrics to comprehensively evaluate the performance of the image restoration model. The peak signal-to-noise ratio (PSNR) and the structural similarity index (SSIM) [53] are used to measure the low-level difference between the restored result and the ground truth. To evaluate IR models from a perceptual perspective, we introduce learned perceptual image patch similarity (LPIPS) [54] and Fréchet inception distance (FID) [55]. These metrics measure the distance between the distribution of the restored images and the ground-truth images. Among them, FID considers more comprehensive feature statistics rather than solely focusing on image quality or diversity, and it is widely used to assess the generative performance of models.

### 4.4. Multiple Degradation Universal Restoration Results

We compared our proposed ETDiffIR with fouruniversal methods, namely, Stable Diffusion (SD) [23], AirNet [17], TKMANet [21], and Universal-IR [18]. These selected methods, as the mainstream methods in the field, ensure the comprehensiveness of the evaluation. Among them, SD and Universal-IR are diffusion-model-based methods, with SD also incorporating text prompts. Specifically, AirNet leverages contrastive learning to obtain degradation features from images and then restores the images through a series of regular convolutions and deformable convolutions [56]. TKMANet utilizes knowledge distillation to learn an universal model from multiple restoration models. Universal-IR is a diffusion-based model, and it shows good performance across multiple datasets. We followed the official experimental settings and retrained these comparative methods on a noisy–rainy–hazy combined dataset.

**Figure 5:**
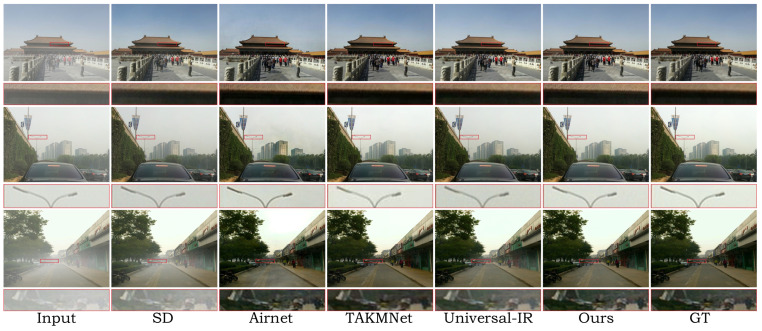
Dehazing comparisons for universal methods on images from the SOTS dataset [51]. The proposed model better preserves image details.

**Figure 6:**
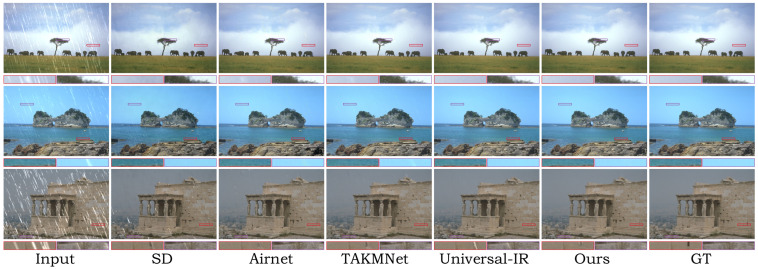
Image deraining comparisons for universal methods on images from the Rain100L dataset [57]. The proposed method effectively removes rain streaks to obtain rain-free images.

**Figure 7:**
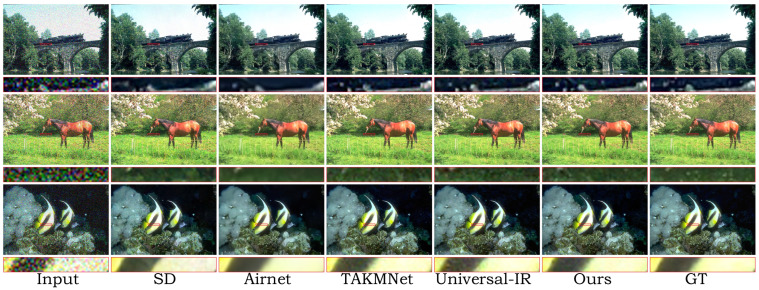
Image denoising comparisons for universal methods on images from the CBSD68 dataset [49].

In Table 1, we report the quantitative comparison results in terms of distortion and perceptual metrics with the state-of-the-art (SOTA) IR approaches in the universal restoration setting. It can be observed that, in the majority of cases, our method outperforms most of the baselines in terms of perceptual metrics, while also showing good performance in terms of distortion metrics. Specifically, our method outperforms the second-best method (Universal-IR), with an average FID improvement of 2.53 across the three degradation types. These results indicate that our method is capable of providing a robust high-quality data distribution for various degradation types, highlighting its strong generative capabilities. Specifically, ETDiffIR outperforms the second-best method (TKMANet) by 1.16 dB in terms of the PSNR on the image dehazing task. From the visual comparisons in Figure 5, it can be observed that our method is able to remove fog at different levels, generating visually pleasing dehazed images. In Table 1, it can be seen that, on the image deraining task, the proposed ETDiffIR provides a substantial gain of 0.8 dB compared to Universal-IR [18]. From the visual comparison in Figure 6, it can be observed that ETDiffIR effectively removes rain streaks, demonstrating strong image reconstruction capabilities. Finally, for the image denoising task, on high-level noise with σ = 50, our method outperforms the Universal-IR [18] method with a significant improvement of 7.06 in terms of FID. Ours-LQ represents the results obtained from testing using captions corresponding to LQ images. When captions corresponding to LQ images are used for restoration, the captions tend to describe the contents of the image less accurately due to the damage in the LQ images. Consequently, all the metrics are slightly inferior compared to the results obtained using the captions corresponding to the HQ images. Figure 7 displays the qualitative comparison results, demonstrating that our method produces denoised images that are more visually pleasing and closer to human perception.

### 4.5. Single Degradation Results

In this section, we evaluate the performance of our ETDiffIR in a single-task setting, training a separate model for each restoration task. We evaluate our model on three distinct degradation tasks. For a more comprehensive evaluation of our model’s performance, we make comparisons using three datasets: we compare image dehazing on the RESIDE-6k dataset [58], image deraining on the Rain100H dataset [59], and image denoising on the CBSD68 dataset [49]. For all three tasks, we compare our method with the prevailing methods in each domain using: GCANet [60], GridDehazeNet [61], and DehazeFormer [62] for image dehazing, and JORDER [63], PReNet [64], and MPRNet [65] for image deraining. We also make a comparison with the advanced multiple-degradation-specific method MAXIM [5] and IR-SDE [31]. Table 2 summarizes the quantitative comparison results on different datasets. On each dataset, our method demonstrates superior performance in terms of perceptual metrics.

### 4.6. Ablation Studies

In Section 4.6.1, we describe the ablation experiments conducted to demonstrate the effectiveness of the guidance generated by the text–image fusion block (TIFB) and efficient restoration UNet (ERUNet). In Section 4.6.2, we analyze why textual prompts can enhance image restoration performance.

#### 4.6.1. Importance of TIFB and ERUNet

To investigate the overall effectiveness of each component in ETDiffIR, we removed the text–image fusion block (TIFB) and efficient restoration UNet (ERUNet) to form the three models reported in Table 3. Baseline represents the model without TIFB and using vanilla UNet for noise prediction. By comparing Baseline and Model-1, we found that EANet demonstrated a 3.4% improvement in terms of FID compared to UNet, while reducing the parameter count and FLOPS by 31.14% and 41.24%, respectively. By comparing Model-1 and our model, we observed that the parameter count of the model increased after adding TIFB, mainly due to the inclusion of the pretrained CLIP text encoder and DA-CLIP image controller in the TIFB. However, the model’s FLOPS only increased by 0.39%, and there was a significant improvement in FID.

**Figure 8:**
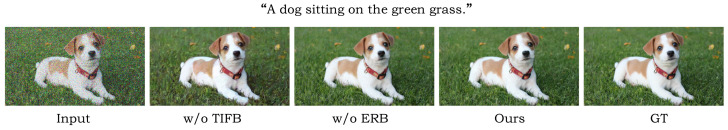
Visualization results of ablation experiments on the effectiveness of the proposed TIFB and ERB.

**Figure 9:**
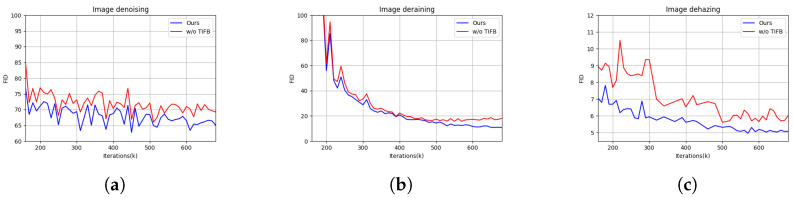
Training curves of model variations, demonstrating the effectiveness of our TIFBs. (**a**) image denoising, (**b**) image deraining, (**c**) image dehazing.

Furthermore, a visual comparison of the models with and without the proposed TIFB is shown in Figure 8. Specifically, the restored images using the TIFB exhibit lower distortion and higher quality. For instance, in the images generated by the TIFB, the color of the grass is more vivid and distinct. Figure 9 illustrates the training curves of our proposed model compared to those of the model without the TIFB in three different image restoration tasks: image denoising, image deraining, and image dehazing. It can be observed that our model’s training is significantly superior.

#### 4.6.2. Effect of Text Prompts

**Figure 10:**
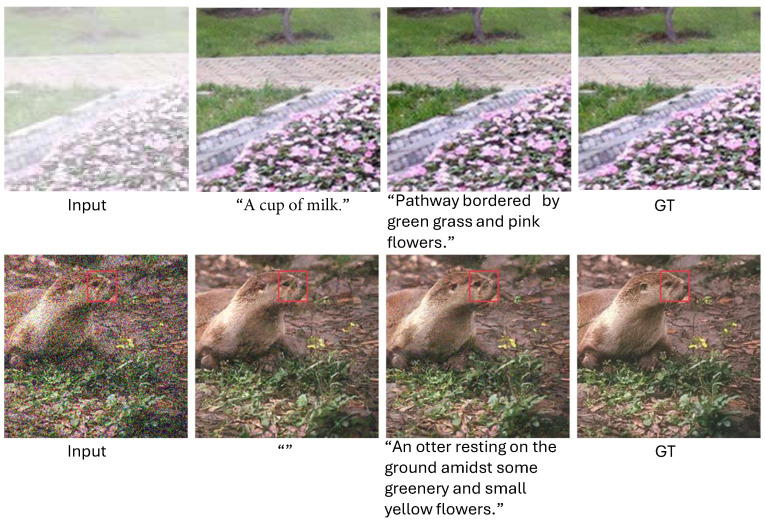
Visual comparison of different textual prompts.

In Figure 10, we compare the restoration results using different captions. As shown, replacing captions with empty text or using inappropriate captions leads to poor image details, while appropriate prompts yield better restoration results. For the upper example, using incorrect captions (i.e., “a cup of milk”) results in insufficient dehazing. For the lower example, using captions results in more accurate details compared to not using captions. This is because text prompts can provide the model with high-level semantic features. These results confirm the effectiveness of text prompts.

## 5. Discussion

The development of deep-learning-based image restoration methods has been rapid and has achieved good results. Due to the potential severe loss of information in low-quality images, the development of image restoration methods is limited. In this paper, we analyzed the limitations of diffusion models in image restoration tasks and proposed a text-guided diffusion model to overcome these limitations in an universal image restoration task. We conducted comparative experiments between the proposed method and other state-of-the-art methods. The experimental results indicate that our model demonstrates improved performance in image restoration on multiple tasks. Additionally, we anticipate the potential of using text prompts to assist image reconstruction in obtaining restoration strategies that are more in line with human visual perception.

The TIFB in ETDiffIR is designed to incorporate text prompts to assist in image restoration. The ERUNet in ETDiffIR is designed to combine text prompts and predict noise conditionally. Specifically, the TIFB utilizes a pretrained CLIP text encoder to encode the textual description corresponding to the image. This encoding is then fused with the degradation encoding obtained from the pretrained DA-CLIP image controller to generate a prompt. Finally, we use this prompt, along with a cross-attention mechanism, to assist the denoising network in obtaining satisfactory results. In the ERUNet, the efficient restoration module extracts global information for each channel and efficiently integrates spatial and channel features. We conducted related ablation experiments to elucidate the functionalities of the TIFB and ERUNet. The related experiments indicate that removing the TIFB and ERUNet leads to an increase in FID, demonstrating the importance of these two components for image restoration.

However, our work still has some limitations. As shown in Table 1 and Table 2, the proposed method achieved competitive results across various metrics for the deraining and dehazing tasks. However, for image denoising, the proposed method performed poorly in terms of the distortion metrics, PSNR and SSIM. Following previous work [18], we set the noise σ value to a challenging 50. While using a lower noise σ value would indeed result in better distortion metrics, denoising large noise is more valuable for research. As a universal restoration model, our model needs to consider the universality of restoration, taking into account the characteristics of other restoration tasks. Additionally, another reason is that the diffusion process is difficult to recognize from Gaussian noise. When using diffusion models for image restoration, additional noise introduced from the Wiener process makes it difficult for the model to distinguish between the Gaussian noise that needs to be restored and the noise from the diffusion process in image denoising tasks. Additionally, due to the iterative sampling required by the diffusion model, our method lacks real-time capabilities. In this paper, we only used synthetic datasets to train the model and did not train it on real degraded datasets. Therefore, the proposed model may require additional optimization for specific use cases.

## 6. Conclusions

In this paper, we designed an effective diffusion probabilistic model guided by text to recover visually pleasing high-quality images from low-quality images. To achieve this, we introduced a text–image fusion module to fully exploit textual information. The text–image fusion block (TIFB) utilizes a pretrained CLIP text encoder to embed textual descriptions corresponding to the images. These embeddings are then fused with image embeddings provided by a pretrained DA-CLIP image controller. Additionally, the proposed efficient restoration U-shaped network (ERUNet) demonstrates superior performance in noise prediction compared to vanilla UNet. Our extensive experiments demonstrate that our proposed method is competitive with state-of-the-art approaches.

## Figures and Tables

**Table 1:** Quantitative comparison with state-of-the-art models in the universal restoration setting: a single universal model is trained on a combined image dataset derived from different degradation types.

Method	Denoising (CBSD68)				Deraining (Rain100L)				Dehazing (SOTS)			
	**PSNR**↑	**SSIM**↑	**LPIPS**↓	**FID**↓	**PSNR**↑	**SSIM**↑	**LPIPS**↓	**FID**↓	**PSNR**↑	**SSIM**↑	**LPIPS**↓	**FID**↓
SD [23]	20.13	0.476	0.286	109.64	26.21	0.712	0.094	32.62	25.49	0.805	0.098	22.40
AirNet [17]	**28.00**	**0.797**	**0.209**	88.22	34.90	0.968	0.028	19.03	27.94	0.962	0.056	18.39
TKMANet [21]	23.94	0.556	0.275	95.68	34.83	**0.970**	0.021	13.10	30.38	0.957	0.047	8.84
Universal-IR [18]	24.36	0.579	0.269	75.03	35.28	0.968	0.017	11.78	30.04	0.962	0.038	5.57
Ours-LQ	25.30	0.641	0.261	70.46	36.01	0.966	0.016	11.61	31.21	0.964	0.036	5.46
Ours	25.84	0.653	0.252	**67.97**	**36.08**	0.969	**0.016**	**11.55**	**31.54**	**0.968**	**0.034**	**5.38**

**Table 2:** Quantitative comparison with state-of-the-art IR models in single-task setting on RESIDE-6k, Rain100H, and CBSD68 test sets. The best value is highlighted in **bold**, while the second-best value is underlined.

Dataset	Method	PSNR ↑	SSIM ↑	LPIPS ↓	FID ↓
Dehaze	GCANet [60]	26.59	0.935	0.052	11.52
	GridDehazeNet [61]	25.86	0.944	0.048	10.62
	MAXIM [5]	29.12	0.932	0.043	8.12
	DehazeFormer [62]	30.29	**0.964**	0.045	7.58
	IR-SDE [31]	25.25	0.906	0.060	8.33
	Ours	**30.44**	0.934	**0.027**	**5.37**
Deraining	JORDER [63]	26.25	0.835	0.197	94.58
	PReNet [64]	29.46	0.899	0.128	52.67
	MPRNet [65]	30.41	0.891	0.158	61.59
	MAXIM [5]	30.81	0.903	0.133	58.72
	IR-SDE [31]	**31.65**	0.904	0.047	18.64
	Ours	31.35	**0.907**	**0.038**	**14.75**
Denoising	CBM3D [66]	24.66	0.675	0.467	144.48
	DnCNN [67]	**28.01**	0.802	0.221	87.23
	FFDNet [68]	27.97	0.789	0.244	98.76
	SUNet [69]	27.88	**0.804**	0.223	68.76
	IR-SDE [31]	25.54	0.689	0.219	97.95
	Ours	26.28	0.695	**0.213**	**63.71**

**Table 3:** Ablation studies of the proposed method. The best performance is shown in **bold**. Note that the metrics are the average results on the CBSD68, Rain100H, and RESIDE-6k datasets.

Method	TIFB	ERBUNet	UNet	Param. (M)	GFLOPS	PSNR ↑	SSIM ↑	LPIPS ↓	FID ↓
Baseline	✗	✗	✓	48.98	129.02	27.66	0.787	0.133	31.11
Model-1	✗	✓	✗	33.73	75.81	28.54	0.798	0.135	30.04
Model-2	✓	✗	✓	174.18	129.52	28.86	0.841	0.085	28.76
Ours	✓	✓	✗	158.93	76.31	**29.36**	**0.845**	**0.093**	**27.94**

## Data Availability

The code will be made available at https://github.com/Iceeteeea/ETDiffIR (accessed on 1 June 2024).
